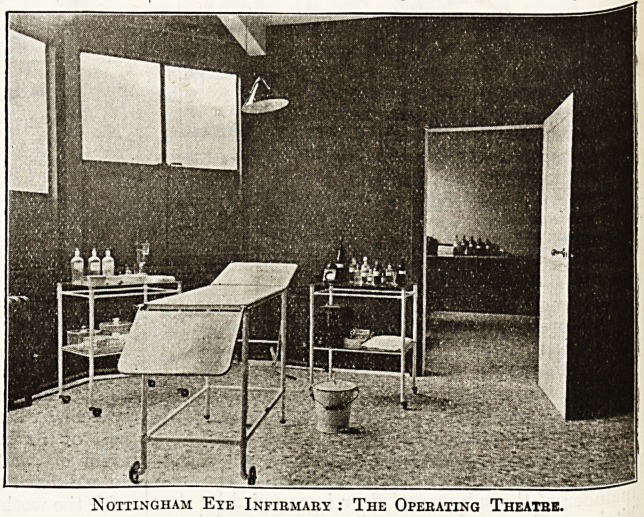# The New Eye Infirmary in Detail and Illustration

**Published:** 1912-07-27

**Authors:** 


### The New Eye Infirmary in Detail and Illustration.

The work of the Nottingham and Midland Eye Infirmary is now carried on in the new premises, thanks to the enterprise of the committee over which Alderman J. P. Ford has presided for 21 years. The following is a slightly abridged description of the building :—

Nottingham Eye Infirmary : The Kitchen.

"The peculiar and irregular site upon which the infirmary stands, with a fall of 18 feet, presented considerable difficulties in planning. A central corridor leads direct to the male wards, and midway between, on the north side, are the operating, sterilising, and clinical rooms. The walls of the former are black—indeed the whole question of lighting for patients undergoing operations has been carefully considered.

"The day-rooms are designed to be utilised as open-air balconies; by a system of patent sliding windows each room can in a few seconds be converted into a sun balcony. Each ward is fitted with a glow light for night use on a new system, by means of which one switch only is used for giving a light of various degrees of strength, from the faintest glow to the full strength—a most useful feature in a hospital ward. The roofs, which are flat and covered with Val de Travers asphalte, are utilised as airing courts for males and females, and shelters are provided for each. The whole of the staff is accommodated on the floor above the wards. There are a servery or kitchen, nurses' dining and sitting room, matron's sitting room, and nurses' and servants' bedrooms, each with their separate bathrooms, lavatories, and conveniences. The floors are all of reinforced concrete. The floor surfaces are of such materials as are durable and non-absorbent and easily cleaned, and there are no corners in the building which are not rounded.

"Each ward has its own sanitary annexe and fire-escape staircase. The sanitary work, water supply, and drainage possess several novel features, all soil pipes are lined with glass, and all drains are cast-iron with porcelain lining. No earthenware pipes are used. There are inspection chambers at every intersection and bend.

"The large out-patient department is entered on the lower level from Oxford Street. A stepless entrance leads into the waiting-hall, which is capable of accommodating about 100, and is fitted with specially designed oak seats. It is a spacious and lofty hall, ventilated by means of an electric fan. A marble fountain is built in the wall at one end. Two doors lead directly from this hall into the large consulting room, equipped with dark-room, microscopic-room, and a dispensary, by which the patients pass out after examination.

"The service entrance is also approached from Oxford Street, where a special yard is provided for the delivery of coals, food supply, laundry, etc., and where the general business requirements are attended to. The kitchen, scullery, larder, meat store, etc., are adjacent to the service entrance.

"The whole of the Rope Walk level is taken up by in-patients and casualty departments. The two women's wards are capable of being sectionally darkened by a system of blinds and shutters, features to which very careful consideration has been given. The colouring of the wards and the glass is green. A boiler in the basement supplies 176 gallons of water per hour at 160° Fahrenheit. The building is heated with low-pressure hot water by radiators of a special hospital pattern devised by the architect, Mr. Arthur Marshall, A.R.I.B.A."

Nottingham Eye Infirmary : The Operating Theatre.

## Figures and Tables

**Figure f1:**
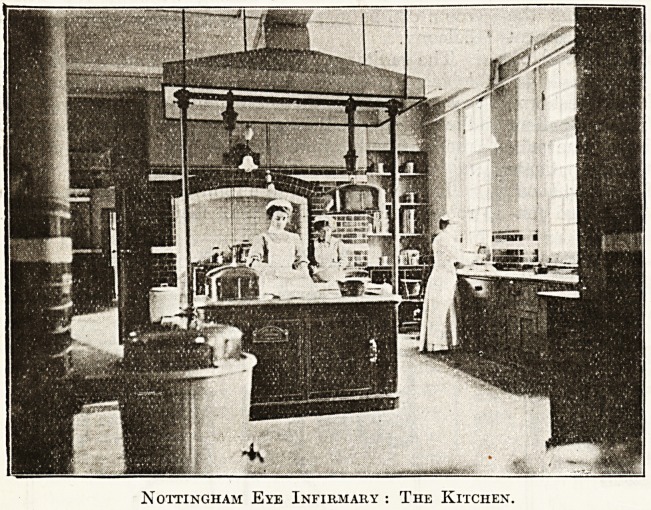


**Figure f2:**